# Population Pharmacokinetics of Primaquine in the Korean Population

**DOI:** 10.3390/pharmaceutics13050652

**Published:** 2021-05-03

**Authors:** Woo-Yul Lee, Dong-Woo Chae, Choon-Ok Kim, Sang-Eun Lee, Yee-Gyung Kwak, Joon-Sup Yeom, Kyung-Soo Park

**Affiliations:** 1 Department of Pharmacology, Yonsei University College of Medicine, Seoul 03722, Korea; mdmartinlee@yuhs.ac (W.-Y.L.); DONGY@yuhs.ac (D.-W.C.); 2 Brain Korea 21 Plus Project for Medical Science, Yonsei University, Seoul 03722, Korea; 3 Clinical Trials Center, Severance Hospital, Yonsei University Health System, Seoul 03722, Korea; 4 Korea Centers for Disease Control and Prevention, Cheongju 28159, Korea; ondalgl@korea.kr; 5 Department of Internal Medicine, Inje University Ilsan Paik Hospital, Goyang 10380, Korea; philmed202@hanmail.net; 6 Department of Internal Medicine, Yonsei University College of Medicine, Seoul 03722, Korea; JOONSUP.YEOM@yuhs.ac

**Keywords:** primaquine, pharmacokinetics, CYP2D6, carboxy-primaquine, malaria, population pharmacokinetics

## Abstract

While primaquine has long been used for malaria treatment, treatment failure is common. This study aims to develop a population pharmacokinetic model of primaquine and its metabolite, carboxyprimaquine, and examine factors influencing pharmacokinetic variability. The data was obtained from a clinical study in 24 Korean subjects randomly assigned to normal and obese groups. The participants received primaquine 15 mg daily for 4 days and blood samples were collected at day 4. Pharmacokinetic modeling was performed with NONMEM and using simulations; the influences of doses and covariates on drug exposure were examined. A minimal physiology-based pharmacokinetic model connected with a liver compartment comprehensively described the data, with CYP450 mediated clearance being positively correlated with the body weight and CYP2D6 activity score (*p* < 0.05). In the simulation, while the weight-normalized area under drug concentration for primaquine in the obese group decreased by 29% at the current recommended dose of 15 mg, it became similar to the normal weight group at a weight-normalized dose of 3.5 mg/kg. This study has demonstrated that the body weight and CYP2D6 activity score significantly influence the pharmacokinetics of primaquine. The developed model is expected to be used as a basis for optimal malaria treatment in Korean patients.

## 1. Introduction

Malaria is a serious disease that affects approximately 228 million people per year worldwide, which caused 405,000 deaths in 2018 [1,2]. Malaria is caused by Plasmodium parasites, which are introduced into the bloodstream through the saliva of an infected mosquito. There are five Plasmodium species that are malariogenic to humans (*P. falciparum*, *P. vivax*, *P. malariae*, *P. ovale*, and *P. knowlesi*). *P. vivax* and *P. ovale* differ from the other species in that they frequently cause relapsing disease [3,4]. In South Korea, 500–600 cases of malaria are reported each year, primarily caused by P. vivax [1].

Primaquine (PQ), an 8-amino-quinoline derivative, is commonly used for radically curing malaria caused by *P. vivax* as part of a combination regimen comprising hydroxychloroquine (administered for 3 days to kill schizonts) and PQ (administered for 14 days to kill hypnozoites) [5]. PQ is also used for primary prophylaxis or prevention of relapse of *P. vivax*—caused malaria, as it is active against hypnozoites in the liver [6].

Although PQ-based treatment is generally successful in curing *P. vivax* malaria, there have been reports of obese individuals exhibiting relapse after the completion of the recommended treatment regimen [7,8,9]. As the anti-parasitic effects of PQ depend on the patient’s exposure to the drug, the most likely cause of this treatment failure is insufficient PQ exposure [7,8,9]. In this respect, it has been suggested that the dose should be adjusted in obese patients [10], however, there have been limited reports of dose adjustment in such patients.

**Figure 1:**
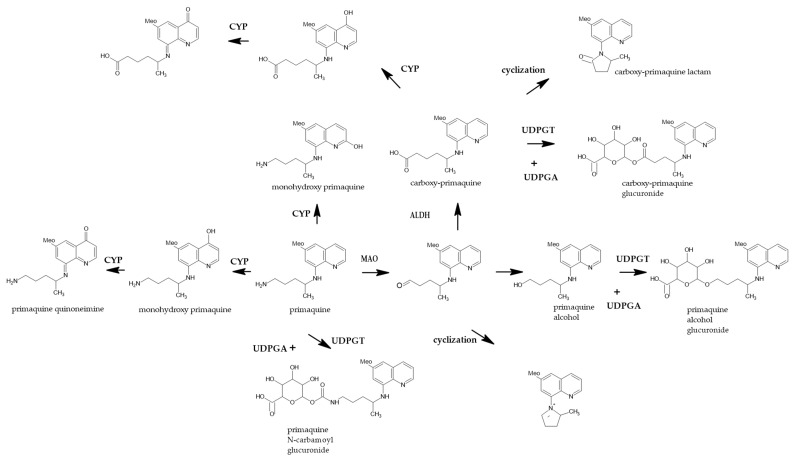
Putative pathways of PQ metabolism in human hepatocyte. ALDH, aldehyde dehydrogenase; CYP, cytochrome P450 (mainly, CYP2D6); UDPGA, Uridine-diphosphate-glucuronic acid; UDPGT, Uridine-diphosphate glucuronosyltransferase. (Adapted from ref. [13], Copyright © Fasinu et al., 2016).

Primaquine is metabolized mainly in the liver by several enzymatic pathways, as shown in Figure 1. The polymorphisms in cytochrome P450 (CYP)2D6 phenotypes are known to affect the outcome of antimalarial treatment, especially in poor metabolizers [11]. Because the active metabolites generated via this pathway are unstable and difficult to measure, the information on their PK profiles and pharmacodynamic relationship is currently unavailable. Therefore, inferences about the PK characteristics of active metabolites cannot but be made on the basis of the PQ dose and CYP2D6 metabolic status [12]. Another enzymatic pathway for primaquine metabolism is the monoamine oxidase (MAO) pathway, which produces the main metabolite carboxyprimaquine (CPQ), whose anti-malarial effect is not fully known.

So far, there has been no pharmacokinetic (PK) analysis of PQ in Asian adult populations. In this respect, the aim of this study is to develop a population pharmacokinetic (PK) model of PQ and to investigate the factors affecting its PK profile. We expect that the developed model may be used as supporting evidence for individualizing PQ dosing. 

## 2. Materials and Methods

### 2.1. Subjects

The data was obtained from a prospective, open-label, parallel design clinical trial. This study was approved by the institutional review board of Severance Hospital, Seoul, Korea (IRB NO. 4-2017-0377) and carried out in accordance with the Declaration of Helsinki. Written informed consent was obtained from each subject before their enrollment in the study. Twenty-four male subjects considered to be healthy on the basis of their medical history, physical examination, 12-lead electrocardiography, and laboratory tests were enrolled in the study. Selection criteria were age 19–50 years and a body mass index (BMI) of 18.6–31.2 kg/m^2^. Exclusion criteria included a medical or surgical history that may have potentially affected the PK of the study drug which was assessed by a physician and clinical pharmacist, clinically significant hypersensitive reaction to hydroxychloroquine or 4-aminoquinolone derivatives, glucose-6-phosphate dehydrogenase (G6PD) deficiency, episodes of primaquine side effects such as hemolytic anemia, methemoglobinemia, and leukopenia, and a history of using any concomitant medications including health supplements and oriental herbal medicine that may pose any interaction with the study drug. To investigate the effects of BW on the PK parameters of PQ, participants with BMI 18.5–24.9 kg/m^2^ were assigned to the normal BW group, and the obese group included subjects with BMI ≥ 25.0 kg/m^2^. The classification of weight by BMI in adult Asians was proposed by a statement from the World Health Organization in 2000 [14,15].

### 2.2. PK Data

Participants received the drug in the manner recommended for administration of the radical cure regimen for P. vivax malaria. Hydroxychloroquine (2000 mg, base) was administered in combination with 15 mg PQ, orally as follows: For the first 3 days, concomitant administration of 800 mg hydroxychloroquine and 15 mg PQ on day 1, followed by 400 mg hydroxychloroquine 10 h thereafter, and 400 mg hydroxychloroquine co-administered with 15 mg PQ on days 2 and 3. On day 4, 15 mg PQ was administered alone and blood samples for PK analysis were collected before drug administration, and 0.5, 1, 1.5, 2, 3, 4, 6, 8, 10, 12, and 24 h after administration (total of 274 blood samples).

### 2.3. Genetic Data

In addition, samples were collected on day 1 for genetic analysis of CYP2D6 (GenBank accession nos. M33388) polymorphisms associated with the production of active PQ metabolites. CYP2D6 genotyping was performed by a commercial laboratory (SPMED Co., Ltd., Busan, Korea). A total of 17 allelic variants were identified in CYP2D6 via gene sequence amplification using polymerase chain reaction (PCR) followed by single nucleotide primer extension (2D6*2, 2D6*3, 2D6*4, 2D6*5, 2D6*6, 2D6*9, 2D6*10B, 2D6*14, 2D6*17, 2D6*18, 2D6*21, 2D6*29, 2D6*41, 2D6*49, 2D6*52, 2D6*60, and 2D6*XN). Subject phenotypes were assigned according to the standard definition of enzyme metabolic activity based on CYP2D6 [16,17,18] and were assigned a phenotype numerical score (activity score (AS) model A) using both alleles from a subject’s genotype [19].

### 2.4. Bioanalysis

PQ and CPQ concentrations were analyzed using plasma samples via a validated liquid chromatography method (Agilent 1200 series; Agilent Technologies, Santa Clara, CA, USA), coupled with mass spectrometry (API 3200; ASICX, Concord, Ontario, Canada; electrospray ionization in positive ion mode). Mass spectrometry operational parameters were as follows: transition (*m/z*) 260→175 for PQ and 275→257 for CPQ; declustering potential 26 for PQ and 16 for CPQ; collision energy (V) 22 for PQ and 20 for CPQ; ion spray voltage 5500 V; source temperature 600 °C. A 200 μL plasma sample was prepared using protein precipitation by mixing the sample with 2 μL 8-aminoquinoline (10 μg/mL) and 400 μL acetonitrile. After centrifugation at 13,000 rpm for 10 min, 5 μL supernatant was injected into a Phenomenex Kinetex C18 column (4.6 × 50 mm, 2.6 µm; Phenomenex, Torrance, CA, USA). The mobile phase comprised 5 mM ammonium formate in acetonitrile with 0.1% formic acid and was delivered using a gradient at a flow rate of 0.5 mL/min. The lower limit of quantification was 1 ng/mL, and precision and accuracy were <15% of the coefficient of variation. The calibration curves showed adequate linearity (r^2^ > 0.99) over a sample concentration range of 1–1000 ng/mL.

### 2.5. PK Model Development

Using a mixed effect model framework, a model parameter was formulated as:

P_i_ = θ × e^ƞi^ (1)

where P_i_ is the parameter value of individual i, θ is the typical parameter value, and η_i_ is a random interindividual difference following a Gaussian distribution with mean zero and variance ω^2^. The residual variability was formulated as:

Y_ij_ = PRED_ij_ × (1 + ε_proij_) + ε_addij_ (2)

where Y_ij_ and PRED_ij_ are the observed and predicted concentrations for individual i at time point j, respectively, and ε_proij_ and ε_addij_ denote proportional and additive residual error, assumed to be normally distributed with mean zero and variance σ^2^_pro_ and σ^2^_add_, respectively. Structural model development then proceeded in two ways, (1) conventional PK (CPK) model and (2) minimal physiology-based PK (PBPK) model.

#### 2.5.1. CPK Model

Blood concentration data for PQ and CPQ were simultaneously fitted in the model. Assuming first-order kinetics for drug elimination, various models were tested for drug absorption and distribution. This included first-order absorption and transit compartment absorption with and without a first pass effect for oral absorption of PQ, and one- and two-compartment models for the distribution of PQ and CPQ. The volume of distribution for CPQ was fixed to 1 L because of parameter identifiability issues.

#### 2.5.2. Minimal PBPK Model

As a semi-mechanistic approach, based on a previous study [20], we considered separately estimating two different metabolic clearance pathways for PQ, the CYP450 (primarily CYP2D6) pathway and the monoamine oxidase (MAO) pathway. While developing the model, the plasma concentrations of PQ and CPQ were not converted to their molar equivalents because they did not differ significantly (259.35 g/mol and 274.31 g/mol, respectively) [21,22]. The liver plasma flow rate was fixed to 49.5 L/h, which is the value estimated assuming a hematocrit level of 45% and liver blood flow rate of 90 L/h [23]. The liver volume (mL) for each subject was also estimated using an equation reported in the literature as 21.585 × BW (kg)^0.732^ × height (cm)^0.225^ [24].

#### 2.5.3. Covariate Analysis

A covariate search was conducted using a stepwise covariate model-building approach based on the likelihood ratio test, with selection criteria of *p* < 0.05 for forward addition and *p* < 0.01 for backward deletion. For continuous covariates such as body weight, age, and CYP2D6 activity scores, relationships between covariate and model parameters were tested with both linear and exponential functions.

### 2.6. Model Evaluation and Simulation

The final model was selected on the basis of various indices, including physiological plausibility, NONMEM objective function value, precision of parameter estimates, and goodness-of-fit diagnostics. The final model selected was then evaluated using a visual predictive check (VPC) by creating 1000 simulated datasets and comparing the model predicted values with the observations. 

For the simulation analysis, plasma concentrations for virtual subjects were generated using the final model for various covariates values, based on the universal PQ dose of 15 mg for 14 days or the optimal dose individualized by using the model. The resulting drug exposure was then examined using the area-under-the-curve (AUC) of a dosing interval at steady state.

### 2.7. Software

Model building was done in NONMEM ver. 7.3 (ICON Development Solutions, Ellicott City, MD, USA) using the first-order conditional estimation with interaction method. Covariate model building and VPCs were performed in Perl-speaks-NONMEM (PsN ver. 4.9.0) and Xpose 4 (ver. 4.0) in R (ver. 3.5.2; R Foundation for Statistical Computing, Vienna, Austria).

## 3. Results

### 3.1. Subject Characteristics

The demographic characteristics and CYP2D6 genotypes of the subjects are shown in Table 1. The mean BMI of the subjects in the obese group was significantly higher than that in the normal weight group (27.8 versus 21.9 kg/m^2^). There were no other significant differences with respect to demographic characteristics between the two groups. CYP2D6 activity score (AS) did not differ significantly between the groups (*p* > 0.05), and none of the patients showed an AS of 0 or above 2.0.

### 3.2. Model Development

#### 3.2.1. Base Model Development

##### CPK Model (Conventional PK Model)

**Figure 2:**
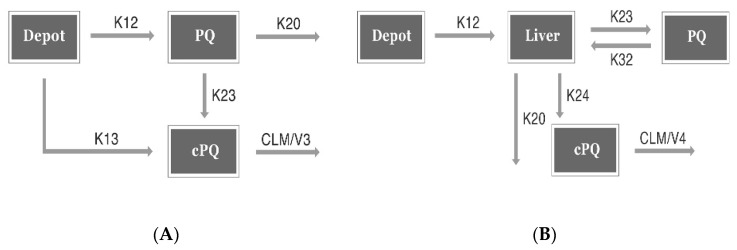
Schematic model. (**A**) Conventional population pharmacokinetics (PPK) model. K12 = (1 − Fa) × KA, K13 = Fa × KA, K23 = FMET × (CL/V2), K20 = (1 − FMET) × (CL/V2), K30 = CLM/V3. PQ, primaquine; CPQ, carboxyprimaquine; KA, absorption rate constant; FMET, fraction of conversion from PQ to CPQ; Fa, 1st pass effect; CLM, clearance of CPQ; V2, volume of distribution for PQ; V3, volume of distribution for CPQ; (**B**) Minimal physiology-based PK (PBPK) model. K12 = KA, K23 = QH × (1 − EH)/V2, K32 = QH/V3, K24 = CL_MAO_/V2, K20 = CL_CYP_/V2, K40 = CLM/V4. PQ, primaquine; CPQ, carboxyprimaquine; KA, absorption rate constant; CL_CYP_, clearance of PQ via CYP2D6 pathway; CL_MAO_, clearance of PQ via MAO (monoamine oxidase) pathway; CLM, clearance of CPQ; EH, hepatic extraction ratio; QH, liver blood flow; V2, liver volume; V3, volume of distribution for PQ; V4, volume of distribution for CPQ. ‘Depot’ denotes the absorption compartment.

A one-compartment disposition model with first-order absorption described the data comprehensively, and incorporating first pass effects significantly improved the model (*p* < 0.001). The schematic diagram for this model is shown in Figure 2A.

##### Minimal PBPK Model (Minimal Physiology Based PK Model)

Using a one-compartment disposition model with first-order absorption as in the CPK model, the incorporation of a liver compartment enabled us to separately estimate the two metabolic clearance pathways for PQ (CYP450 and MAO), as well as to model the CPQ formed by MAO. The theoretical allometric approach using body weight did not improve the model, thus it was not applied to the base model. A schematic diagram of this model is shown in Figure 2B. According to our model diagnostic criteria, the minimal PBPK model showed similar model performance as the CPK model. The minimal PBPK model was chosen as its parameters for metabolites were physiologically more meaningful.

#### 3.2.2. Final Model Development

Using the minimal PBPK model as a basis, stepwise covariate model building was performed and BW and CYP2D6 AS were found to influence CL_CYP_ significantly (*p* < 0.001) in such a way that

CL_CYP_ = 7.5 L/h × e^(1.25 × (AS − 1.5) + 0.04 × (BW − 77.45))^,

where CL_CYP_ denotes the clearance of PQ via the CYP pathway. Other than BW and AS, no covariate was significant.

The final model showed that CLMAO, the conversion clearance from PQ to CPQ via the MAO pathway, was 19.1 L/h, which is approximately 2.5 times that via CLCYP, and CPQ clearance was estimated to be 1.3 L/h. The volumes of distribution for PQ and CPQ were 142.2 L and 30.1 L, respectively. The model predicted mean values for V3, CLCYP, and CLMAO in the obese group increased by approximately 4.3%, 97.4%, and 0.36%, respectively, compared to those in the normal weight group. The estimated model parameters and their variability are presented in Table 2.

### 3.3. Model Evaluation and Simulation

**Figure 3:**
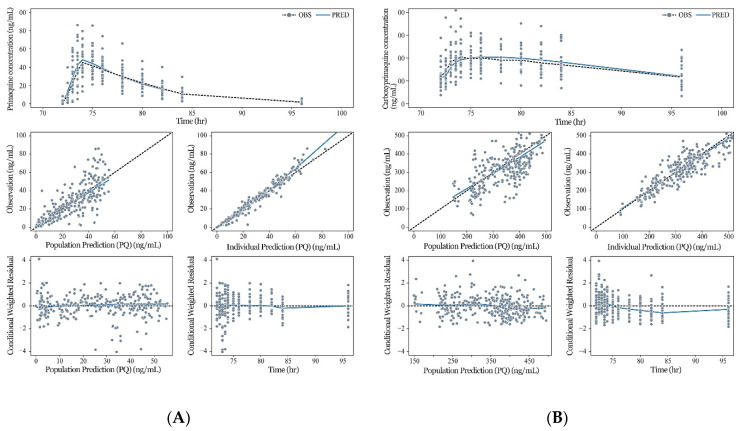
Goodness-of-fit plots for the final PK model for primaquine (PQ) and carboxyprimaquine (CPQ). (**A**) Goodness-of-fit plots for PQ; TOP: Observed concentrations (dots), their smooth (dotted line), and population predictions (solid line) vs time for PQ; MIDDLE: Observed concentrations vs population predictions (left) and vs individual predictions (right) for PQ (solid line: smooth; dashed line: line of identity); BOTTOM: Conditional weighted residual vs population prediction (left) and vs time (right) for PQ (solid line: smooth; dashed line: line of zero residual); (**B**) Goodness-of-fit plots for CPQ; TOP: Observed concentrations (dots), their smooth (dotted line), and population predictions (solid line) vs time for CPQ, MIDDLE: Observed concentrations vs population predictions (left) and vs individual predictions (right) for CPQ (solid line: smooth; dashed line: line of identity); BOTTOM: Conditional weighted residual vs population prediction (left) and vs time (right) for CPQ (solid line: smooth; dashed line: line of zero residual). OBS, observation; PRED, prediction.

**Figure 4:**
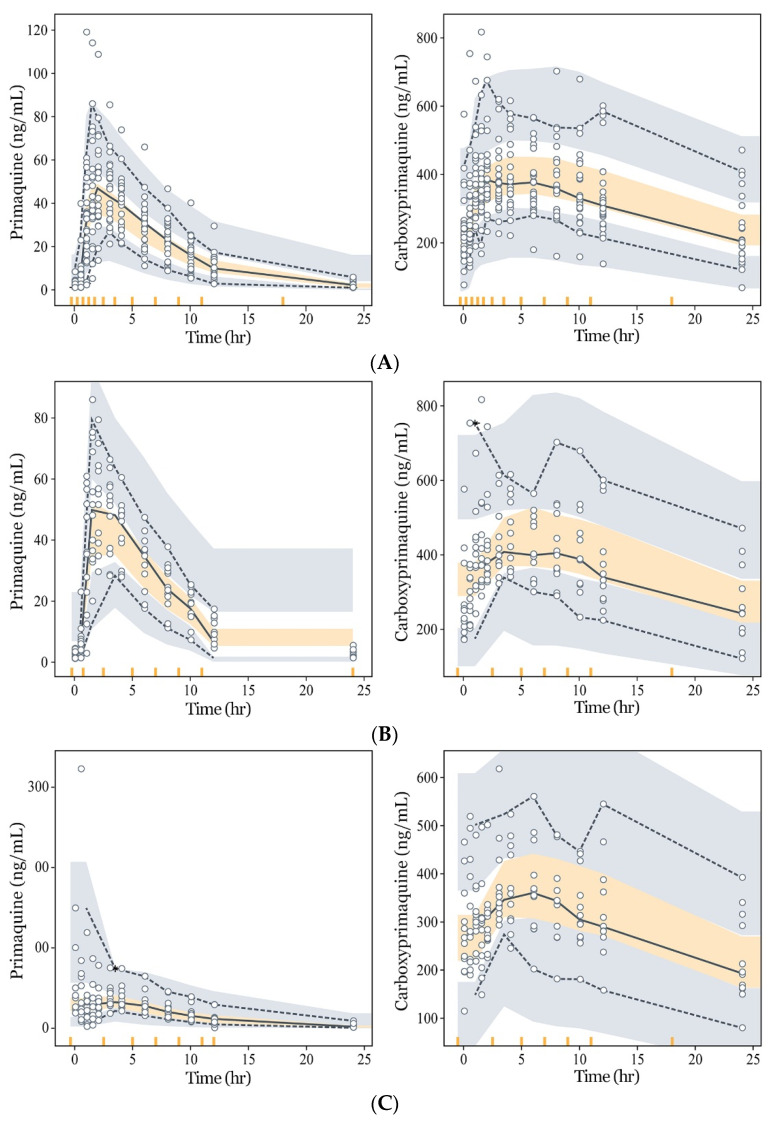
Visual predictive checks (VPC) of the final PK model for primaquine (PQ) (left) and carboxyprimaquine (CPQ) (right) obtained from 1000 simulations (upper dashed line: 95 percentile; middle solid line: 50 percentile; lower dashed line: 5 percentile). (**A**) VPC with data from both groups; (**B**) VPC with data from the normal weight group; (**C**) VPC with data from the obese group.

Goodness-of-fit assessments are presented in Figure 3, and diagnostic VPCs of the model are shown in Figure 4. The PQ and CPQ population prediction curves described the data well, showing no clear systematic bias in the structural and residual error models (Figure 3). VPC plots for the entry data (Figure 4A) showed that most of the observed concentrations fell within the 95% predictive interval of simulated data, suggesting the appropriateness of the final model. VPCs performed separately within each group (Figure 4B,C) and also indicated that the observed concentrations were well within the 95% predictive interval, further suggesting the appropriateness of the model. Furthermore, observed concentrations of PQ and CPQ were lower in the obese group (Figure 4B,C), justifying the use of BW as a covariate in our model.

For simulation analyses, the predicted weight normalized AUC_PQ_ and AUCC_PQ_ after multiple administration of PQ for 14 days generated from the final model were approximately 28.7% and 28.4% lower in the obese group compared to the normal-weight group, respectively. (Table 3). Here, the reason that AUC was normalized with respect to weight is to reflect the influence of weight in comparisons, which was selected to be a significant covariate.

**Figure 5:**
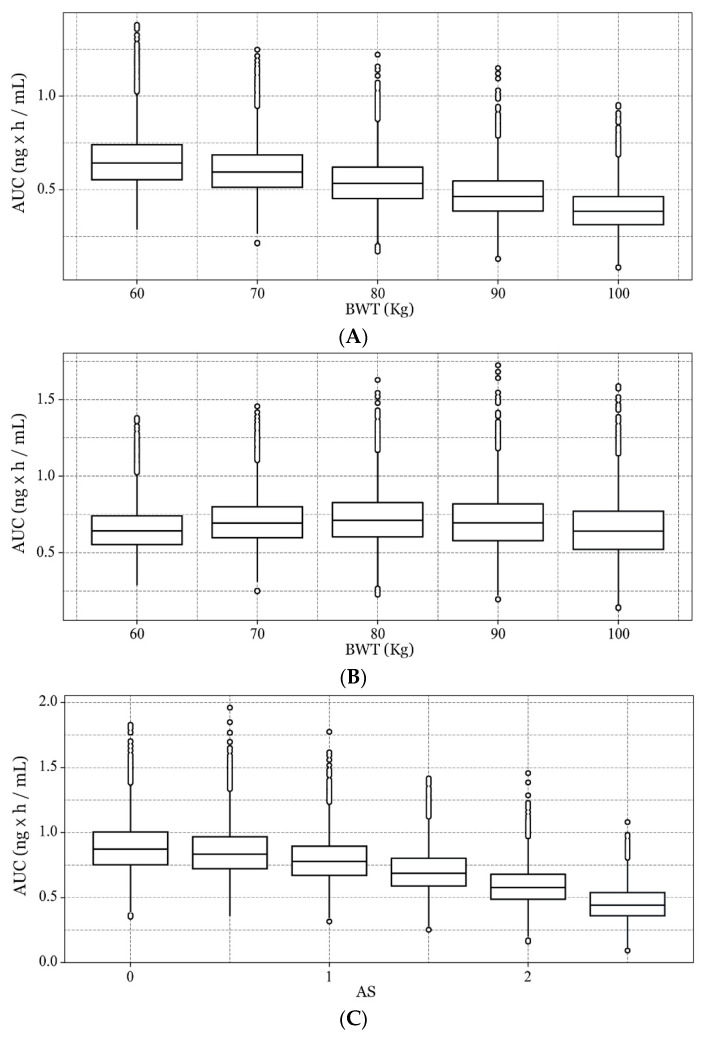
Simulated primaquine (PQ) area-under-the-curve (AUC_PQ_) in groups with different body weights (BWT). (**A**) AUC_PQ_ after administration of a flat dose of 15 mg for the 60 kg, 70 kg, 80 kg, 90 kg, and 100 kg groups; (**B**) AUC_PQ_ after administration of a weight normalized dose of 0.25 mg/kg (15 mg for 60 kg, 17.5 mg for 70 kg, 20 mg for 80 kg, 22.5 mg for 90 kg, and 25 mg for 100 kg); (**C**) AUC_PQ_ versus CYP2D6 activity score (AS) for a dose of 17.5 mg administered to individuals with 70 kg body weight and 175 cm height.

When examining virtual individuals with a BW of 60–100 kg, a median AS of 1.5, and height of 175 cm, the mean AUC_PQ_ decreased by approximately 12% for every 10 kg. In contrast, when administration of a weight-normalized dose of 0.25 mg/kg was considered, the simulated AUC_PQ_ became similar across all BW groups. Furthermore, AUC_PQ_ declined with AS over an AS range of 0 to 2.5 (Figure 5).

## 4. Discussion

In this study, we aimed to investigate the PK of PQ in the Korean population and formulate suggestions for appropriate dose calculations for obese patients. Our data showed that PQ exposure was lower in the obese group than in the normal BW group. This difference in PQ exposure was well explained by our model incorporating BW into the liver volume and PQ clearance. Applying the universal dose of 15 mg to all individuals regardless of BW leads to reduced drug exposure in obese patients, and through simulation using the PK model developed, we showed that administration of weight-normalized doses could achieve similar drug exposure over the entire weight range examined. For different BW groups between 60 and 100 kg that were administered the conventional dose of 15 mg/day for 14 days, the predicted AUCs decreased as the BW increased, showing approximately 40% smaller AUC in the 100 kg group compared to the 60 kg group (Figure 4A). When weight-adjusted PQ doses, 0.25 mg/kg/day, were administered, the simulated AUCs were similar across all BW groups (Figure 4B). Here, the weight was normalized in accordance with a previous study in which the total optimal PQ dose to achieve the maximum therapeutic outcome was suggested to be 3.5 mg/kg [25]. According to this study, the conventional PQ dosage regimen of 15 mg QD was ideal for patients weighing 60 kg, which was equivalent to the weight-adjusted dose of 0.25 mg/kg/day used in our study.

Because the hydroxylated active metabolite cannot be measured accurately and its data were not available, the PK model for PQ in our study was built on the basis of exposure data for the inactive metabolite CPQ, as reported in previous studies [12,26,27]. In those studies, although the PK-PD relationship of the active metabolite was undocumented, the authors made inferences about the PK characteristics of active metabolites based on the PK characteristics of PQ and CPQ [12], insisting that enhanced anti-malarial effectiveness was likely to be related to higher plasma concentrations of PQ [27], and PQ underdosing and subsequent low levels of PQ may contribute to a high risk of recurrent malaria infection [26].

BW was found to be in our study a significant covariate to ensure an adequate exposure level of PQ, which was consitent with a previous result [7,12,28,29]. However, there is a lack of study regarding the influence of obesity on the PK of PQ, and the plausible mechanism for this is currently unknown. Nevertheless, since metabolism via CYPs, except for CYP3A4, and phase II conjugating reactions are obviously increased in the obese population, lower exposure of PQ might be partially explained by increased liver metabolism [30].

**Figure A1:**
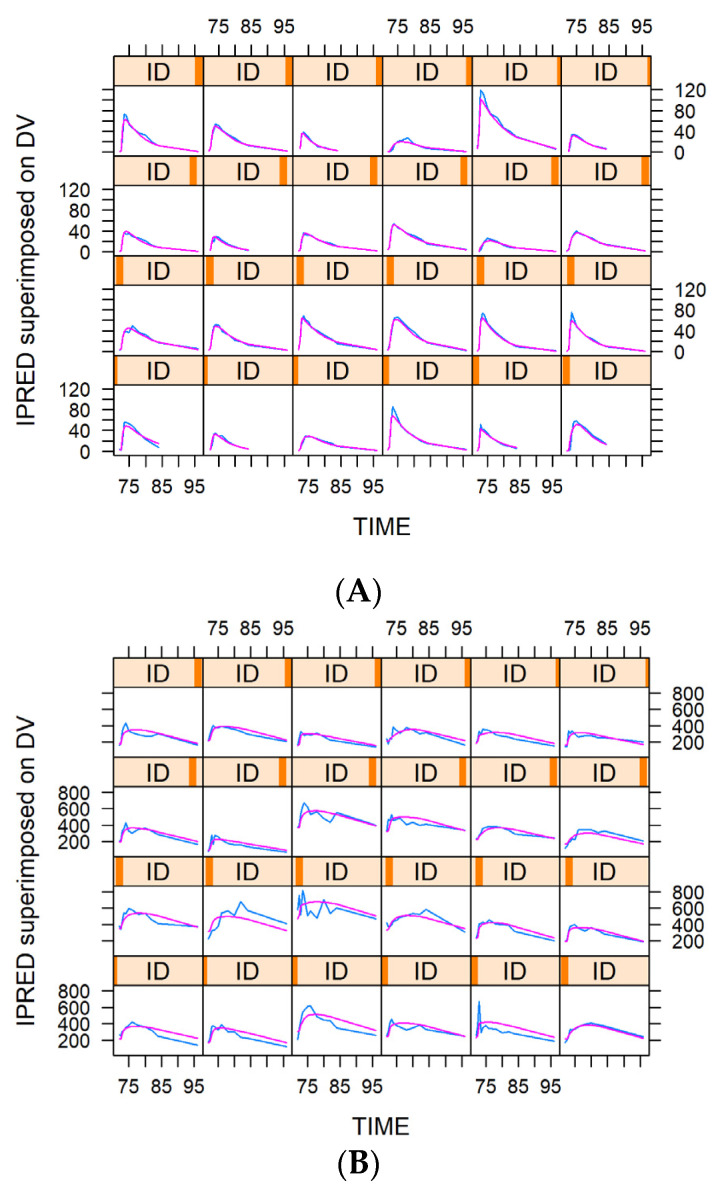
Plots for individual observations (blue) and predictions (red). (**A**) Primaquine, (**B**) Carboxyprimaquine.

In the VPC plots, there is second peak seen in CPQ (metabolite) profiles. For example, in Figure 4, there is a slight increase in the 95 percentile of CPQ at around 10 h. This is seen in many PK studies of PQ [31], which can be explained by (1) accumulation of metabolites that should further metabolize through carboxylation, and (2) late absorption of PQ due to enterohepatic circulation [32,33]. However, the second peaks in Figure 4 contain very few data points, representing only 5% of the observations, and only appeared in the small number of subjects in the individual profiles (see the Appendix A Figure A1), indicating that it is not a significant feature of the PK of the drug.

PQ is primarily metabolized by CYP2D6 and MAOs in the liver [34,35,36,37]. The anti-parasitic effect of PQ is thought to be mostly attributed to the CYP2D6 clearance, which produces hydroxylated metabolites that are believed to exert pharmacological effects via oxidative stress. It is likely that the mechanisms of action of PQ on parasites are mediated by reactive oxygen species produced during the redox cycling of hydroxylated metabolites formed via the CYP2D6 pathway [11,38]. For MAOs, this major metabolic clearance pathway produces CPQ, the main metabolite of PQ. However, the role of CPQ regarding anti-malarial activity is not fully known and its pharmacological effects remain to be elucidated. Unfortunately, the PK and PD characteristics of the active oxidative metabolite are undocumented. Since the PK characteristics of oxidative metabolite probably depend mostly on the PQ dose and CYP2D6 metabolic activity [12], quantifying the two metabolic pathways separately is important. The 2.5-fold larger estimation of CLMAO compared to CLCYP shows that it plays a major role in PQ metabolism. The reason that body weight was incorporated only into CLCYP in the final model is that the estimate of CLCYP increased by approximately 97.4% in the obese group, while that of CLMAO increased by only 0.36%.

In subjects with impaired phenotypes of CYP2D6, the adequate amount of dosing may not provide desirable clinical outcomes due to the low active metabolites generated. Although the pharmacologically active PQ metabolite cannot be measured, which is a considerable drawback, some inferences can be made on the basis of the pharmacokinetic model of the parent drug. In the model developed here, we tried to reflect the effect of CYP2D6 polymorphism by incorporating AS into CLCYP. The metabolism via CYP pathway decreased by approximately 84.7%, 71.3%, and 46.5% in the individuals with AS of 0, 0.5, and 1, respectively, compared to the group with AS of 1.5, which was the modal value of AS in our data. This enables us to adjust the approximate amount of PQ dose required to achieve a similar level of active metabolites in the group with impaired CYP2D6 alleles, to that in the group with the normal range of phenotypes (Figure 4C). While compensating with a higher PQ dose may benefit poor metabolizers [12,39], more clinical studies are needed, since the PK-PD relationship of PQ including active metabolites is not fully known.

There are some differences in our model compared to the previous study [20]. First, age was not included in our model and the BW was incorporated to CLCYP only, rather than being applied to all volume and clearance parameters, since theoretical allometry failed to improve our model significantly. Second, the parameter estimates for CLMAO and VCPQ (volume of distribution for CPQ) were approximately 2.6, and 1.4-fold higher in our study, respectively. These differences could be due to a (1) different age distribution of enrolled subjects (children aged 2–14 years vs adults), (2) ethnic difference (African vs Asian), (3) different PQ formulation and co-administered drugs (solution formulation co-administered with artemether-lumefantrine vs tablet formulation co-administered with hydroxychloroquine). However, the estimated parameters were similar to those from another Asian study conducted in the Thai population in which the combination regimen of chloroquine and primaquine was used [40].

Our study has several limitations. First, all of our study subjects were healthy volunteers, and our model may need to be validated in patients. Second, our exponential covariate model for CL_CYP was derived from the data with severely obese individuals not included. Thus, for extremely obese individuals, say those with BW ranging from 100 to 120 kg, the model can produce CL_CYP as large as 2.5 to 5.5 times that for the typical individual with BW of 77 kg, and one should be careful about interpreting the results for such individuals. For obese individuals, it is known that the lean body weight rather than the total body weight might be a better predictor [41]. Additional study may be needed for validation and more precise dose adjustment for these special cases. Nonetheless, this does not affect the total clearance significantly since PQ is metabolized mainly via the MAO pathway. Third, these preliminary results are from a study with small sample size. To generalize the result, it should be validated in a large group of patients. Fourth, this study is based on the PK profiles of the parent drug and its major but inactive metabolite, rather than its active metabolites. Hence, special consideration is required for individuals with variant CYP2D6 phenotypes. For instance, for the same PQ concentration observed, poor metabolizers would yield lower levels of active metabolites while ultra-rapid metabolizers would yield higher levels of active metabolites. Therefore, for more precise optimal dosage regimen design tailored for different CYP2D6 metabolic activities, additional studies will be needed to identify the relationship between PQ and active metabolite levels. Lastly, this study used the data obtained over the period of only four days of dosing, which might not be long enough to achieve a steady state for CPQ based on the half-life of 24.8 h previously reported [40]. However, from the perspective of the population PK used in our study, this cannot be a limitation.

Nevertheless, to our knowledge, this was the first population PK study for PQ in the Korean adult population, providing a scientific evidence-based rationale for the appropriateness of a weight-based dosing regimen. The results demonstrate that the total PQ dose, body weight, and genetic mutations in CYP2D6 may influence the effective PQ dose and a low dose may lead to treatment failure or a relapse of disease [42]. Although more studies are needed to validate the results in this study, our modeling work provides the basic knowledge for future studies to identify the PK-PD relationship of PQ in malaria. The results here may be applied to the prophylactic use of PQ for individuals visiting malaria-endemic regions, including those of Asia.

## 5. Conclusions

The present study described the population PK model of PQ, quantifying the effect of body weight and CYP2D6 phenotypes on the clearance of PQ. We suggested some evidence about the potential need of weight-based dosing instead of a “one for all size of dose” especially in the obese group. Further studies on the active metabolites and the mechanism of action of PQ are needed to generalize the result.

## Figures and Tables

**Table 1:** Baseline characteristics of the study subjects.

Demographics	Normal Group (*n* = 12)	Obese Group (*n* = 12)	*p* Value *
Male, *n* (%)	12 (100.0)	12 (100.0)	–
Age (years)	29.1 ± 8.4	26.4 ± 7.4	0.417
Height (cm)	175.4 ± 4.6	173.1 ± 5.1	0.251
Body weight (kg)	67.6 ± 7.6	83.3 ± 6.7	<0.001
Body mass index (kg/m^2^)	21.9 ± 2	27.8 ± 1.8	<0.001
CYP2D6, *n* (%)			1.000
AS (= 0.5)	0 (0.0)	1 (4.2)	
AS (= 1.0)	3 (12.5)	3 (12.5)	
AS (= 1.5)	6 (25.0)	6 (25.0)	
AS (= 2.0)	3 (12.5)	2 (8.3)	

Data are presented as means ± SD or as *n* (%). * *p* values for differences between the two groups were calculated using independent t-tests for continuous variables and Fisher’s exact tests for CYP2D6 count comparison. Abbreviations: PM, poor metabolizer; IM, intermediate metabolizer; EM, extensive metabolizer; UM, ultra-rapid metabolizer.

**Table 2:** Final model parameter estimates.

Parameter	Description	Estimate (Unit)	RSE (%)
**Structural Parameters**			
V3	Volume of distribution for PQ	142.2 (L)	3.9
V4	Volume of distribution for CPQ	30.1 (L)	8.4
CL_MAO_	CL of PQ via MAO pathway	19.1 (L/h)	7.5
CL_CYP_	CL of PQ via CYP2D6 pathway	7.5 (L/h)	27.6
CLM	CL of CPQ	1.3 (L/h)	8.7
KA	Absorption rate constant	1.7 (h^−1^)	12.7
ALAG1	Absorption lag time	0.45 (h)	1.7
COVAS	Covariate effect of AS on CL_CYP_	1.254	11
COVBW	Covariate effect of BW on CL_CYP_	0.041	9.1
**Inter-Individual Variabilities**			
ω^2^_V3_	BSV on V3	16.6 (CV%)	13.4
ω^2^_CLMAO_	BSV on CLMAO	22.7 (CV%)	23.9
ω^2^_CLCYP_	BSV on CLCYP	55.2 (CV%)	30
ω^2^_KA_	BSV on KA	82.9 (CV%)	16.1
ω^2^_CLM_	BSV on CLM	20 (CV%)	21.4
ω^2^_ALAG_	BSV on ALAG	6.8 (CV%)	30.6
**Residual Error**			
σ^2^_pro1_	Proportional error of PQ	17.9 (CV%)	13.9
σ^2^_add_	Additive error of CPQ	22.3 (SD)	31.7
σ^2^_pro2_	Proportional error of CPQ	15.7 (CV%)	14.2

Abbreviations: RSE, relative standard error; CL, clearance; PQ, primaquine; CPQ, carboxy-primaquine; MAO, monoamine oxidase; AS, activity score; BSV, between subject variabilities; SD, standard deviation; CV, coefficient of variation.

**Table 3:** Simulated * AUC of primaquine and carboxyprimaquine in the normal and obese groups after administration of a flat dose of primaquine 15 mg for 14 days.

Parameter	Normal Weight Group (*n* = 12)	Obese Group (*n* = 12)	*p* Value
AUC_PQ_ (ng·h/mL)	610.2 ± 149.5	538.6 ± 160.2	<0.01
AUCPQ (ng·h/mL/Kg)	9.2 ± 2.6 **	6.5 ± 2.1 **	
AUC_CPQ_ (ng·h/mL)	8917 ± 2323.4	7903.9 ± 2521.9	<0.01
AUCCPQ (ng·h/mL/Kg)	134 ± 40.2 **	95.9 ± 32.8 **	

Data are presented as means ± SD. AUC_PQ_: predicted area-under-the-curve within a dosing interval of primaquine. AUC_CPQ_: predicted area-under-the-curve within a dosing interval of carboxyprimaquine. * simulation: 15 mg primaquine for 14 days. ** normalized by body weight.

## Data Availability

The datasets used and/or analyzed during the current study are available from the corresponding author on reasonable request.
